# A Four Month Randomized Controlled Trial on the Efficacy of Once-daily Fenofibrate Monotherapy in Persons with Spinal Cord Injury

**DOI:** 10.1038/s41598-019-53753-7

**Published:** 2019-11-20

**Authors:** Michael F. La Fountaine, Christopher M. Cirnigliaro, Joshua C. Hobson, Alexander T. Lombard, Adam F. Specht, Trevor A. Dyson-Hudson, Steven C. Kirshblum, William A. Bauman

**Affiliations:** 1 Department of Veterans Affairs Rehabilitation Research & Development Service National Center for the Medical Consequences of Spinal Cord Injury, James J. Peters Veterans Affairs Medical Center, Bronx, NY USA; 2 Department of Physical Therapy, School of Health and Medical Sciences, Seton Hall University, South Orange, NJ USA; 3 Departments of Medical Sciences and Neurology, Seton Hall-Hackensack Meridian School of Medicine, Nutley, NJ USA; 4 The Institute for Advanced Study of Rehabilitation and Sports Science, School of Health and Medical Sciences, Seton Hall University, South Orange, NJ USA; 5 Department of Kinesiology and Applied Physiology, University of Delaware, Newark, DE USA; 6 Kessler Foundation, West Orange, NJ USA; 7 Department of Physical Medicine and Rehabilitation, Rutgers New Jersey Medical School, Newark, NJ USA; 8 Kessler Institute for Rehabilitation, West Orange, NJ USA; 9 Departments of Medicine and Rehabilitation Medicine, Icahn School of Medicine at Mount Sinai, New York, NY USA

**Keywords:** Outcomes research, Risk factors

## Abstract

An open-label, randomized clinical trial of once-daily fenofibrate monotherapy administered for 2- (Mo2) and 4- (Mo4) months using modified intervention thresholds for triglyceride (TG) was performed in persons with chronic spinal cord injury (SCI). Fenofibrate (145 mg tablet) was self-administered daily in 10 persons with SCI for 4 months with monthly blood testing to quantify the lipoprotein profile (e.g., serum TG, LDL-C, and HDL-C concentrations). Eight SCI participants were control subjects. In comparison to the control group, the treatment group at Mo2 had a 40% (±12%; p < 0.05) reduction in serum TG concentration, a 28% (±21%; p < 0.05) increase in HDL-C and 14% (±20%; p < 0.05) decline in LDL-C. In the same comparison at Mo4, the treatment group maintained a 40% (±20%; p < 0.05) reduction in serum TG concentration, had an 18% in reduction in LDL-C (±12%; p < 0.05) and a 23% (±23%; p < 0.05) increase in HDL-C. Fenofibrate monotherapy for Mo2 and Mo4 initiated in persons with SCI resulted in a robust and favorable change in the serum lipoprotein profile and ratios, suggesting reduced risk for cardiovascular disease.

## Introduction

Persons with spinal cord injury (SCI) have a combination of risk factors for cardiometabolic dysfunction that contribute to a mixed dyslipidemia profile^1,2^ that may include depressed serum high-density lipoprotein cholesterol (HDL-C) concentrations and serum low-density lipoprotein cholesterol (LDL-C) and serum triglyceride (TG) concentrations that are generally in the normal range^3–5^. Many individuals with SCI would not meet the criterion to initiate an appropriate therapeutic agent to modify their lipid profile based on the current clinical practice guidelines^6,7^. The difficulty of adhering to this general therapeutic approach is that there is evidence for a heightened risk of cardiovascular disease related mortality in persons with SCI compared to the general population^8–10^, with some of the heightened risk attributable to the reduction in the levels of HDL-C.

In a prior report, our group demonstrated that functional sympathetic nervous system (SNS) innervation to the liver and abdominal tissues influenced the concentrations of circulating TG-rich lipoproteins in a cohort of non-ambulatory persons with chronic SCI^11^. Individuals with a neurological level of SCI at or proximal to the 4^th^ thoracic vertebrae (↑T4), had significantly lower TG concentrations than individuals with an injury at or distal to the 5^th^ thoracic vertebrae (↓T5)^11^; this vertebral level is important because preganglionic projections begin to emerge from the spinal cord below T4 to innervate the abdominal viscera and adipose tissue through the celiac and superior mesenteric ganglion^12,13^. Thus, because of functional ablation of sympathetic innervation to the viscera, the “normal” serum TG concentrations in persons with higher lesions of the spinal cord may not adequately capture the true risk for cardiovascular disease (CVD) if one were to apply the general guidelines developed to treat dyslipidemias in the general population^6,7^.

To determine if a different threshold value for abnormal serum TG concentrations was present in SCI cohorts, a retrospective analysis was performed in 223 persons with SCI ↑T4 and 178 with SCI ↓T5 to identify the serum TG concentrations that were equal to a serum HDL-C that is identified as being an independent risk factor for coronary artery disease (CAD; e.g., <40 mg/dl)^14^. The serum TG threshold was reported to be lower in persons with SCI (↑T4 ≥ 115 mg/dl and ↓T5 group ≥ 137 mg/dl) than the clinically accepted threshold to consider initiating hypolipidemic therapy in the general population (i.e., TG ≥ 150 mg/dl). Therefore, the opportunity may exist for persons with SCI to benefit from an appropriate pharmacological intervention to reduce the relatively elevated serum TG concentrations.

Fenofibrate is a fibric acid derivative that acts on the peroxisome proliferator-activated receptor α (PPAR-α)^10,15^. Evidence for the efficacy of treatment with PPAR-α agonists has spanned more than 5 decades and has demonstrated reduced serum concentrations of TG and LDL-C and increased serum HDL-C in several clinical trials^16–28^. PPAR-α agonists are often the first line agent persons with hypertriglyceridemia^29^. Despite the volume of available evidence for its efficacy in the general population, no clinical trials have been performed to determine what effect the medication has on the lipoprotein profile of persons with SCI. This study evaluated the efficacy of once-daily fenofibrate administration after 2 and 4 months on changes to the lipoprotein profile in persons with SCI and elevated serum TG concentrations relative to standards used in the general population.

## Results

**Figure 1:**
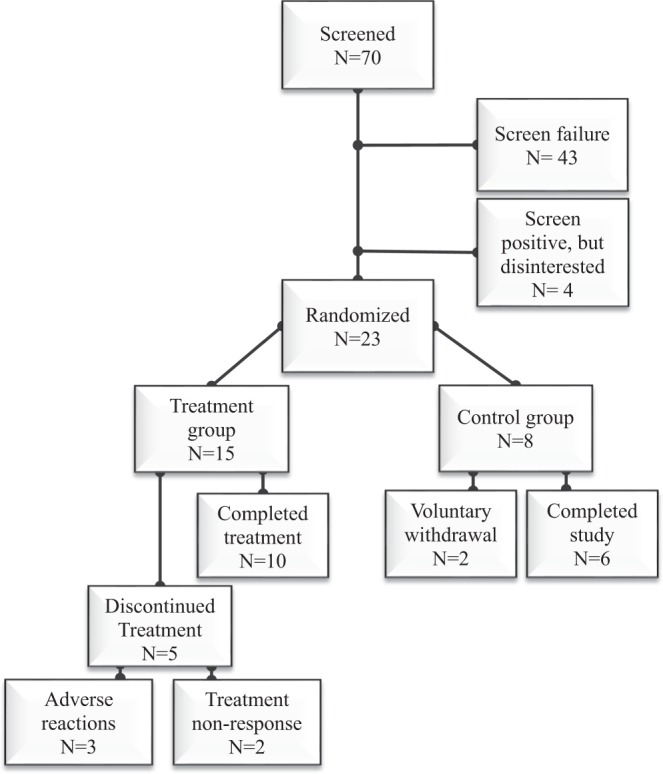
CONSORT diagram of participant engagement in trial.

Seventy persons with SCI met the screening study entrance criteria and 23 participants with elevated serum TG concentrations by study entrance criteria were identified; each participant agreed to be randomized to the treatment or control groups (Fig. 1). In total, 15 participants initiated an open-label, once-daily treatment with fenofibrate and 8 participants served as control subjects. Data from 10 treatment and 8 control subjects who completed the Mo4 trial are presented herein, and the demographics are provided (Table 1). Two control subjects discontinued their participation after the Mo2 visit to pursue a separate clinical trial; the serum laboratory values from their last visit were carried forward to the Mo3 and Mo4 visits. Five treatment subjects were discontinued from study drug. Two participants receiving treatment were discontinued per protocol after Mo2 for not achieving a ≥25% reduction in the serum TG concentration. Three treatment subjects experienced an adverse event (e.g., 1 had elevated LFTs and 2 had abnormal bowel patterns) that began shortly after initiating or after a few weeks of drug treatment. The two subjects who experienced abnormal bowel patterns requested and were granted a discontinuation from the trial. One participant had a drug-related elevation in LFTs (e.g. ≥2.5 times greater than the upper limit of normal) that resulted in termination from the study. A second treatment subject had an elevated LFT at the Mo2 visit which was deemed to be unrelated to the drug treatment (e.g., binge drinking in the days prior to the study visit) with a return to acceptable values after a short period of abstinence; the IRB approved our request for the participant to continue in the study. Apart from these adverse events, there were no other adverse signs or symptoms attributable to treatment with the study drug in any participant during the trial. Data from the safety laboratory values are provided for the 10 treatment and 8 control subjects who completed the 4-month trial (Table 2).

**Table 1:** Characteristics of the Study Groups.

	Control	Treatment	p Value
n	8	10	—
Age (years)	44 ± 13	49 ± 14	NS
Height (m)	1.80 ± 0.07	1.78 ± 0.10	NS
Weight (kg)	82.5 ± 16.8	99.7 ± 30.5	NS
BMI (kg/m^2^)	25.4 ± 5.3	31.1 ± 7.8	NS
DOI (years)	18 ± 15	17 ± 12	NS
Gender (M/F)	8/0	9/1	NS
Paraplegia/Tetraplegia (n)	2/6	6/4	NS
AIS (A/B/C)	6/2/0	4/3/3	NS
Ethnicity (AA/Hisp/White)	0/3/5	2/3/5	NS

Data are presented as group mean ± SD. Abbreviations: AA = Afrian American; AIS = American Spinal Injury Association Impairment Scale; BMI = body mass index; DOI = duration of injury; F = female; Hisp = Hispanic; NS = not significant.

**Table 2:** Safety Outcomes for Each Visit by Group.

	Normal Range	Baseline		Month 1		Month 2		Month 3		Month 4	
		Control	Treatment	Control	Treatment	Control	Treatment	Control	Treatment	Control	Treatment
n		8	10	8	10	8	10	8	10	8	10
eGFR	>59	126 (107, 144)	121 (106, 135)	125 (105, 144)	117 (103, 130)	124 (97, 150)	114 (103, 124)	127 (95, 159)	112 (101, 120)	121 (89, 153)	115 (100, 131)
ALT	0–32 IU/L	21 (15, 28)	21 (17, 26)	24 (10, 38)	37 (13, 60)	23 (12, 32)	35 (20, 50)	19 (11, 28)	33 (19, 46)	21 (14, 27)	32 (19, 45)
AST	0–40 IU/L	20 (15, 25)	22 (19, 25)	22 (11, 33)	27 (19, 36)	23 (9, 36)	29 (19, 40)	20 (13, 28)	25 (20, 30)	18 (13, 23)	28 (21, 36)
GGT	0–60 IU/L	27 (16, 37)	27 (17, 36)	24 (14, 34)	40 (17, 80)	25 (17, 33)	31 (12, 51)	24 (15, 33)	33 (19, 48)	25 (12, 39)	22 (14, 30)
RBC	3.77–5.28 g/dL	4.7 (4.3, 5.1)	4.8 (4.3, 5.1)	4.8 (4.2, 5.3)	4.7 (4.4, 5.1)	4.8 (4.3, 5.2)	4.6 (4.3, 5.0)	4.7 (4.2, 5.2)	4.6, (4.3, 5.0)	4.9 (4.4, 5.4)	4.5 (4.2, 4.8)
WBC	3.4–10.8 × 10e^3^/uL	6.3 (4.6, 7.9)	6.5 (5.4, 7.7)	6.5 (5.4, 7.7)	6.2 (5.0, 7.4)	6.9 (3.2, 10.5)	6.8 (5.0, 8.6)	6.3 (2.7, 10.0)	6.3 (4.7, 7.8)	7.1 (3.5, 10.7)	6.0 (4.2, 7.7)
MCV	79–97 fL	90 (86, 94)	86 (82, 90)	90 (87, 92)	87 (84, 90)	90 (87, 93)	87 (84, 89)	90 (87, 93)	87 (84, 90)	90 (87, 93)	87 (84, 89)
MCH	26.6–33.0 pg	30.4 (29.1, 31.6)	29.3 (28.2, 30.6)	30.4 (30.0, 31.2)	29.2 (28.0, 30.4)	30.2 (29.3, 31.0)	29.1 (28.0, 29.8)	30.5 (29.5, 31.5)	28.9 (28.0, 29.8)	30.2 (29.2, 31.1)	29.3 (28.4, 30.2)
MCHC	31.5–35.7 g/dL	33.8 (33.2, 34.4)	34.3 (33.7, 34.9)	33.9 (33.3, 34.5)	33.6 (33.2, 34.0)	33.5 (33.2, 33.9)	30.6 (23.7, 37.4)	33.9 (33.1, 34.7)	32.8 (31.7, 33.9)	33.5 (33.1, 34.0)	33.8 (33.2, 34.3)
RDW	12.3–15.4%	13.8 (13.2, 14.4)	14.3 (13.8, 14.8)	13.8 (13.0, 14.5)	13.8 (13.0, 14.5)	13.8 (13.0, 14.5)	14.5 (13.8, 15.0)	13.9 (13.3, 14.5)	14.3 (13.8, 15.0)	13.9 (13.4, 14.3)	14.8 (14.0, 15.5)

Data are expressed as group mean ± 95% CI, unless otherwise indicated. eGFR: estimated glomerular filtration rate; ALT: alanine aminotransferase; AST: aspartate aminotransferase; GGT: gamma-glutamyl transferase; RBC: red blood cell; WBC: white blood cell; MCV: mean corpuscular volume; MCH: mean corpuscular hemoglobin; MCHC: mean corpuscular hemoglobin concentration; RDW; red cell distribution width.

Even with the 2:1 randomization, the groups were matched at baseline for serum TG, HDL-C, VLDL-C, and plasma glucose and insulin concentrations; the treatment group had a significantly greater serum LDL-C and TC concentrations than the control group (p < 0.05; Table 3). For each serum lipid value (e.g., TG, HDL-C, LDL-C, VLDL-C and TC), RMANOVA revealed the presence of a significant time main and interaction effect across the primary study time points (e.g., baseline, Mo2, Mo4). The serum HDL-C was significantly higher at Mo2 (p < 0.01) and Mo4 (p < 0.05) in the treatment group compared to the control group (Table 3). The serum TG and VLDL-C concentrations trended toward being significantly lower at Mo2 (p = 0.09), and Mo4 (p = 0.06) in the treatment group compared to the control group, respectively (Table 3).

**Table 3:** Blood and Lipid Profiles for Each Visit by Group.

	Baseline		Month 1		Month 2		Month 3		Month 4		p value		
	Control	Treatment	Control	Treatment	Control	Treatment	Control	Treatment	Control	Treatment	Group	Visit	Interaction
n	8	10	8	10	8	10	8	10	8	10	—	—	—
Triglyceride (mg/dl)	189 ± 75	204 ± 70	178 ± 76	128 ± 41	166 ± 78	120 ± 45*	156 ± 58	145 ± 60	183 ± 79	122 ± 55^*^	NS	0.01	0.001
HDL-C (mg/dl)	35 ± 7	38 ± 8	37 ± 10	47 ± 8	36 ± 10	49 ± 9‡	35 ± 6	46 ± 9	35 ± 7	47 ± 12^†^	0.05	0.01	0.05
LDL-C (mg/dl)	107 ± 20	131 ± 20^†^	115 ± 23	114 ± 23	115 ± 30	111 ± 28	113 ± 25	111 ± 21	46 ± 13	106 ± 16	NS	0.01	0.05
VLDL-C (mg/dl)	38 ± 15	41 ± 14	36 ± 15	26 ± 8	33 ± 16	24 ± 9^*^	31 ± 12	29 ± 12	37 ± 16	25 ± 11^§^	NS	0.001	0.01
Total Cholesterol (mg/dl)	180 ± 20	210 ± 30^†^	188 ± 31	187 ± 29	185 ± 34	184 ± 40	180 ± 31	186 ± 24	176 ± 21	177 ± 28	NS	0.01	0.05
Glucose (mg/dl)	88 ± 14	86 ± 11	88 ± 6	90 ± 10	87 ± 9	91 ± 6	91 ± 13	84 ± 8	86 ± 8	87 ± 7	NS	NS	NS
Insulin (mIU/ml)	20 ± 15	12 ± 10	16 ± 16	19 ± 13	11 ± 7	12 ± 7	14 ± 8	14 ± 10	18 ± 21	12 ± 8	NS	NS	NS

Data are expressed as group mean ± SD. HDL-C: high-density lipoprotein cholesterol; LDL-C: low-density lipoprotein cholesterol; VLDL-C: very low-density lipoprotein cholesterol. ^*^p = 0.10; ^†^p < 0.05; ^‡^p < 0.01; ^§^p = 0.06.

**Figure 2:**
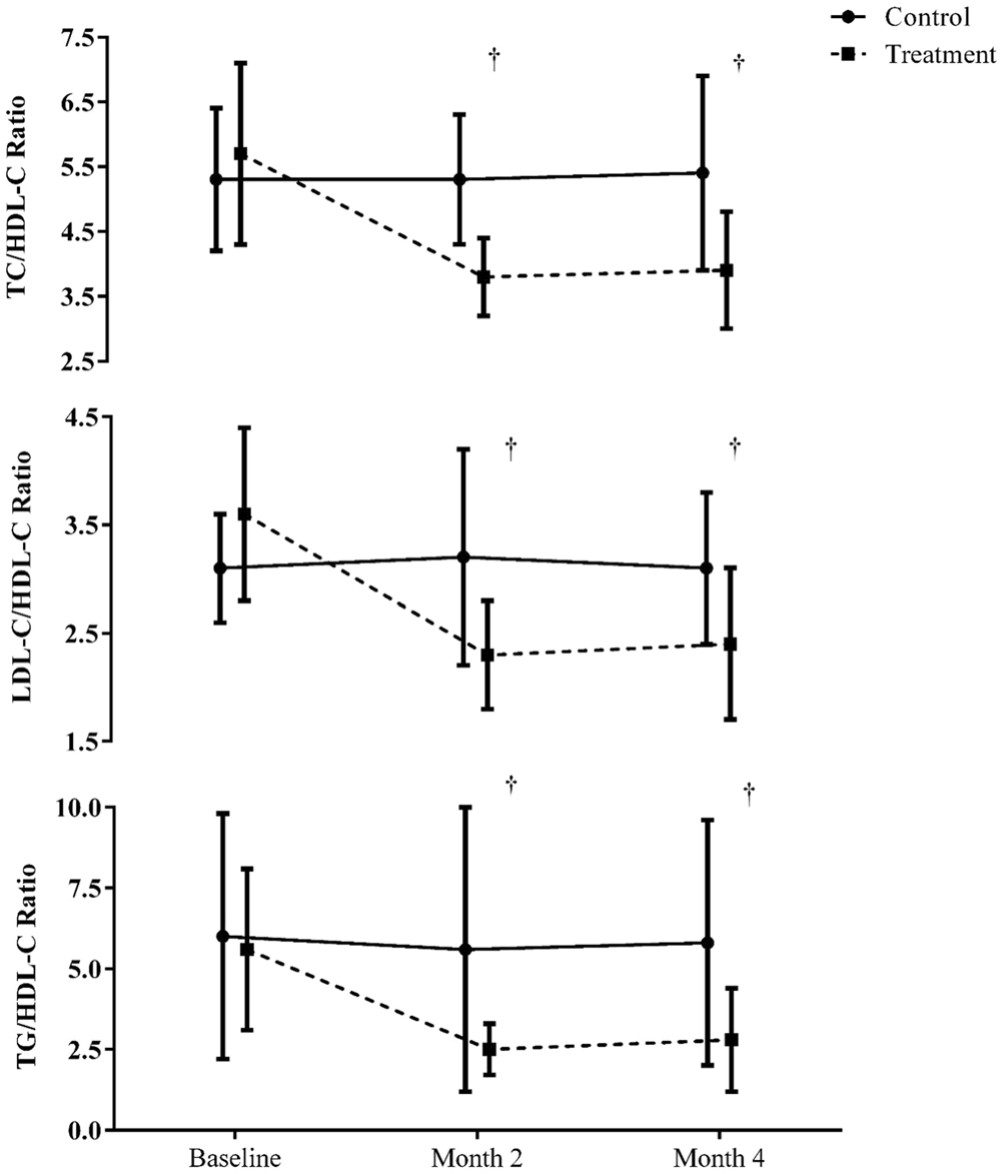
Changes in the TG/HDL-C, LDL-C/HDL-C and TC/HDL-C ratios by group at each visit. Data are offset to enhance visualization. A significant time main (p < 0.01) and interaction (p < 0.01) effect were observed for each ratio. Post-hoc tests reveal that the Month 2 and 4 values in the treatment group were significantly lower than controls (^†^p < 0.05).

The TG/HDL-C, LDL-C/HDL-C and TC/HDL-C ratios were not significantly different between groups at baseline. A significant time main and interaction effect was observed across the primary study time points (e.g., baseline, Mo2, Mo4). Post hoc tests revealed that the treatment group had significantly lower ratios at Mo2 and Mo4 (p < 0.05) than the control group (Fig. 2). By Mo4 in the treatment group, the TG/HDL-C ratio decreased 50% from 5.6 to 2.8, the LDL-C/HDL-C ratio decreased 32% from 5.6 to 3.7, and the TC/HDL-C ratio decreased 30% from 3.5 to 2.4. No significant change in the lipid value ratios was noted in the control group.

**Figure 3:**
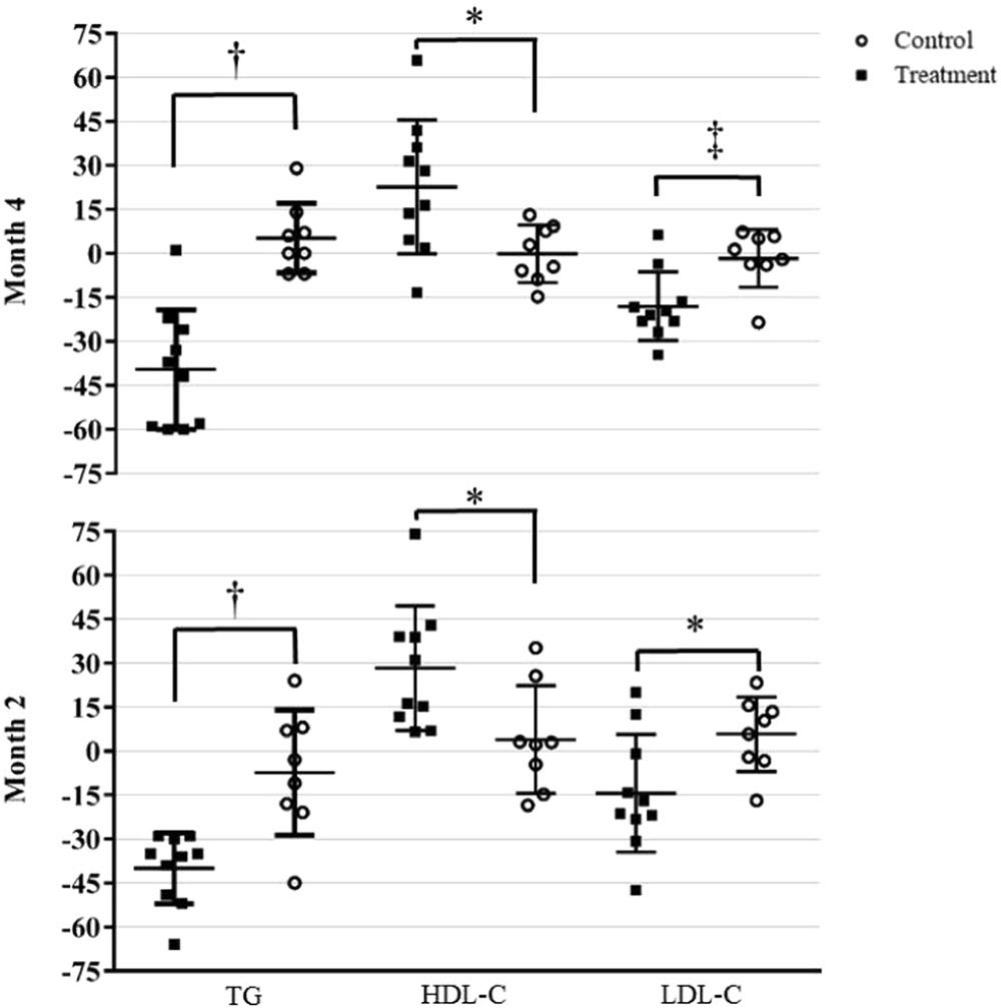
Percent change from baseline for lipids by group. Data presented as dot plots with the group mean (horizontal line) and standard deviation for each lipid. For each within visit comparison of a lipid, the groups had significantly different percentage change from baseline (^†^p < 0.001; ^‡^p < 0.01; *p < 0.05).

Because of the inherent variability of lipid concentrations between participants, the percent change from baseline to Mo2 and Mo4 was calculated for serum TG, HDL-C, and LDL-C concentrations. For each lipid at Mo2 and Mo4, the treatment group had a significantly different percent change from baseline compared to the control group (Fig. 3). After Mo2, the treatment group had an average 40% (±12) reduction in serum TG, a 28% (±21) increase in serum HDL-C and 14% (±20) decline in serum LDL-C concentrations, whereas the control group had 8% (±21) reduction in serum TG, a 4% (±18) increase in serum HDL-C and 6% (±13) increase in serum LDL-C concentrations. Compared to baseline values, at Mo4 the treatment group maintained a 40% (±20) reduction in the serum TG concentration, had a further reduction in serum LDL-C concentration to 18% (±12), and had a further increase in the serum HDL-C concentration to 23% (±23). At Mo4, the control group had 5% (±12) increase in serum TG, a 2% (±13) decrease in serum LDL-C and 0% (±18) change in serum HDL-C concentrations (Fig. 3).

## Discussion

This is the first study in persons with SCI to test the effect of once-daily fenofibrate monotherapy to modify the lipoprotein profile after 4 months of treatment. The intervention had a robust effect to produce favorable changes to the serum TG, HDL-C and LDL-C concentrations with relatively few side/adverse effects. The study design had a SCI-centric TG threshold concentration for screening and potential study inclusion, which was based on our prior reports that describe the effect of functionally impaired SNS innervation to the abdomen; higher spinal cord lesions were observed to lower circulating concentrations of TG-rich lipoproteins^11^. By prior statistical modeling, the TG concentration that intersects with serum HDL-C concentration of 40 mg/dl is lower in persons with SCI than that of the general population^30^. The threshold values for pharmacological intervention in this clinical trial were lower than that recommended by conventional guidelines for hypolipidemic therapy in the general population^6,7^. Of note, approximately half of our treatment cohort had baseline serum TG concentrations <150 mg/dl.

Treatment with fenofibrate (or other PPAR-α agonist equivalents) in persons with hypertriglyceridemia has shown a therapeutic efficacy to promote favorable changes in the serum TG (41–53% decrease), LDL-C (6–20% decrease), and HDL-C (5–20% increase) concentrations in clinical trials of varied durations, clinical endpoints, and patient populations with adverse lipid concentrations^16–23^. These combined changes are the results of the many actions of this class of drugs on the liver and other internal organs. PPAR-α, a ligand-activated transcriptional factor that binds to a DNA sequence in tissues where mitochondrial and peroxisomal fatty acid β-oxidation rates are relatively high^15,31^, such as in the brown fat, heart, kidney, liver and skeletal muscle^31^, increasing the mobilization of fatty acids^32^, enhancing β-oxidation and upregulation of apolipoprotein A-I and AII synthesis in the liver^32–34^, an effective inhibition of fatty acids by the liver, and increased lipoprotein lipase activity^35^.

Therapy with fibric acid derivatives can reduce serum TG concentrations between 41–53% and increase HDL-C between 5–20%^16–28^. It would be reasonable to expect that the greatest percentage change occurs in those individuals with the most abnormal baseline concentrations at the start of the intervention. In our treatment cohort, about half of the subjects had baseline serum TG concentrations <150 mg/dl and the remaining subjects had values that were between 207 and 298 mg/dl; of note, the effective percent change in serum TG concentrations after Mo4 intervention was not different, regardless of the baseline serum TG value. In clinical trials using fenofibrate in the general population where the group mean serum TG concentrations were between 128–277 mg/dl at baseline, a range that encompasses the majority of participants in our treatment group, an average 26% reduction in serum TG concentration was observed at the final study time point^16,17,23–28^. In a subset of those trials with able-bodied participants who had a group mean HDL-C between 37–47 mg/dl at baseline^17,23,24,26,28^, the effective percent increase in HDL-C at the conclusion of treatment was only ~12%. In our treatment group, there was a 40% decrease in the serum TG concentration and a 28% and 23% increase in serum HDL-C at Mo2 and Mo4, respectively. These findings exceeded what may be expected as an interventional change to the lipid profile while on study drug among individuals in the general population with normal or slightly elevated serum TG concentrations at the start of the intervention and provide support that persons with SCI may have a lower threshold value to consider drug intervention to reduce risk for CVD than that of the general population^11,30^. For unclear reasons, one treatment participant who was assumed to be 100% compliant with the study medication—that is, the participant did not return any unused study drug—had serum TG and HDL-C concentrations return to baseline levels at Mo4; if the data of this subject had been excluded from our analyses, the mean serum TG and HDL-C concentrations for the treatment group at Mo4 would have been even more favorable, with values of 44% and 28%, respectively.

One of the most consistently abnormal lipid anomalies in persons with SCI is a low serum HDL-C concentration^4^. In one study in veterans with SCI, 24% had serum HDL-C values between 30–34 mg/dl and 13% had values were less than 30 mg/dl^36^. The cause of the serum HDL-C reduction is multifactorial and likely due to varying degrees of insulin resistance, obesity, and lifestyle choices (e.g., physical activity volume, diet preference). In our trial we observed a 28 and 23% increase in serum HDL-C at Mo2 and Mo4, respectively; this favorable change in serum HDL-C highlights the well-documented inverse relationship between circulating TG molecules and HDL-C particles. Elevated serum TG concentrations are capable of depleting cholesterol within a HDL-C particle through a two-step process in which TG replaces cholesterol ester in the lipid core in a one-to-one ratio through the action of cholesterol ester transfer protein; the TG in the HDL-C core is then hydrolyzed by lipases, resulted in a cholesterol-depleted HDL particle of reduced size^37^. This smaller HDL-C particle is then thought to be more readily metabolized and/or excreted by the kidney^38,39^, contributing to a lower circulating concentration of serum HDL-C. Based on our findings, the effect of fenofibrate treatment to lower the TG concentrations may be speculated to have reduced the hydrolysis of the HDL particle, thus permitting the serum concentrations to rise during the treatment period.

There is an on-going discussion regarding the benefit of lowering serum TG concentrations to reduce the risk of CVD-related mortality, despite the presence of several randomized clinical trials showing a significant benefit for a range of study endpoints^40^, including a decreased risk of non-fatal myocardial infarctions^41^. In a long-term follow-up study^21^ on mortality in patients who completed the Helsinki Heart Study^19,20^, the group that received study drug had a 32% lower relative risk of CAD mortality compared to the placebo group, and after further follow-up, the relative risk was 23% lower (i.e., 18 years after completing the initial study)^21^. An interesting aspect from this secondary analysis revealed that subjects in the highest body mass index and serum TG levels tertiles (of the study cohort) who received the study drug had a 71% lower relative risk of CAD mortality and a 33% lower relative risk of all-cause mortality compared to the placebo group^21^. The authors concluded that treatment with gemfibrozil, a fibric acid derivative that is appreciated to have comparable therapeutic effects to fenofibrate^32^, reduced long-term mortality in patients with dyslipidemia and the metabolic syndrome^21^. The findings of enhanced efficacy in those with dyslipidemia and the metabolic syndrome, or diabetes mellitus, have been reproduced and further expanded to include a decreased presentation of CAD by angiography^22^. The TG/HDL-C, the LDL/HDL-C and TC/HDL-C ratios are recognized potent discriminators of risk for atherosclerosis (e.g., TG/HDL-C ratio >4.0)^42^, CAD^43,44^, and ischemic heart disease^45^. At baseline, 6/10 subjects had a TG/HDL-C ratio >4.0. Whereas at Mo4, 9/10 had a ratio <4.0, and the group mean TG/HDL-C ratio had decreased 50% at Mo4. The treatment group also had reductions in the LDL-C/HDL-C and TC/HDL-C ratios that corresponded to a reduction in the odds-ratio for ischemic heart disease from of 1.9 to 1.0 and 2.6 to 1.0, extrapolating from data of the Quebec Cardiovascular Study^45,46^. Although by design our trial was relatively short, and there were no long-term goals of this study, the absolute changes in the lipoprotein profile, as well as the several calculations of risk, highlight a robust effect of fenofibrate treatment at Mo4 in a SCI cohort in which little empirical data has been reported to modify lipid factors related to CVD morbidity and mortality.

In the only other clinical trial performed in persons with SCI aimed at modifying the lipoprotein profile, extended-release niacin (nicotinic acid) was administered in an escalating dose fashion for 48 weeks^47^. The study found a dose-effect that resulted in ~13% and ~14% decrease in the serum TG and LDL-C concentrations, respectively and a 25% increase in the serum HDL-C by the end of treatment. These changes had a corresponding effect to lower the TC/HDL-C ratio by 26% and the LDL-C/HDL-C ratio by 32% at the end of the 48-week trial (the TG/HDL-C ratio was not calculated). In our clinical trial, the absolute salutary changes in serum lipid values had the effect to lower the TC/HDL-C ratio by 30%, the LDL-C/HDL-C ratio by 32%, and a 48% decrease in the TG/HDL-C ratio.

This was a small-scale open-label clinical trial in persons with SCI designed to assess the safety and efficacy of once-daily fenofibrate monotherapy for 4 months. The health of persons with SCI may frequently deteriorate, which introduces challenges that are not applicable to clinical trials that are performed in the general population. These challenges contributed to our small sample size, but did not interfere with the successful completion of subjects who received the treatment intervention. The study sites were in a single geographical region of the country and the services available to persons with SCI in the New York City metropolitan area may be more extensive than those that are accessible in other regions of the country, or available in other countries. As a result, the clinical presentation and lifestyle choices of our participants may not adequately represent or be generalizable to the SCI population that lives outside of a major metropolitan area. Females represent ~20% of the entire SCI population, but our study cohort had only had 1 female subject in the treatment group (10%), making it difficult to make any meaningful inferences on the role of gender in our study outcomes or to discuss any implications of the treatment with fenofibrate on the health of women with SCI.

In conclusion, four months of fenofibrate monotherapy initiated in persons with SCI at a lower serum TG concentration than that which triggers therapeutic pharmacological intervention in the general population resulted in a 40% and 18% reduction in serum TG and LDL-C concentrations, respectively, as well as a 23% increase in serum HDL-C with few adverse side effects. The adverse events observed were among those that have been previously reported with fenofibrate administration^10,48^, including events related to the digestive system in persons with SCI who have a well appreciated high prevalence of bowel dysfunction and, as such, may warrant special consideration. The findings from this clinical trial in persons with SCI should serve as proof-of-concept and as an impetus to perform future investigations in which the effect of different classes of hypolipidemic agents may be compared for safety and efficacy. A larger clinical trial of longer duration is necessary to better determine the efficacy of fenofibrate in a heterogeneous SCI population.

## Methods

### Study cohort

A prospective, open-label efficacy trial was performed in persons with chronic SCI to determine if once-daily fenofibrate initiated at serum TG concentrations (i.e., paraplegia: ≥135 mg/dl; tetraplegia ≥115 mg/dl)^30^ that are lower than what is conventionally performed in the general population, led to favorable changes in the serum lipoprotein profile after 2 and 4 months of treatment, respectively. To be considered for study enrollment, subjects must have met the inclusion criteria [e.g., male or female between the ages of 21 and 69 with American Spinal Injury Association Impairment Scale (AIS) designation of A, B, or C]^49^ and exclusion criteria [e.g., having acute illness or infection; having reduced glomerular filtration rate (e.g., eGFR < 60 ml/min) or liver function tests (LFTs: ≥2.5 times greater than the upper limit of normal); current pharmacological treatment with agents known to effect the serum TG concentration; hypersensitivity to fenofibrate; existing CAD, congestive heart failure, or recent history of myocardial infarction (i.e., ≤12 months); pregnancy or women who may become pregnant during the course of the study, or those who are nursing; have diminished mental capacity; and/or an inability or unwillingness of subject to provide informed consent].

### Procedures

A screening visit was performed in persons with SCI to identify subjects with elevated serum TG concentrations (i.e., paraplegia: ≥135 mg/dl; tetraplegia ≥115 mg/dl) after a ≥12-hour overnight fast. Participants identified as having elevated serum TG values were randomized in a 2:1 manner to receive once-daily fenofibrate therapy (145 mg tablet; Tricor^®^, AbbVie Inc., North Chicago, IL, USA) or be control subjects for 4 months. According to standard clinical practice guidelines, if after 2 months (Mo2) of treatment with fenofibrate, a ≥25% reduction in the serum TG concentration was not observed, drug therapy was discontinued. For those who responded to fenofibrate therapy (e.g., a reduction of serum TG ≥ 25%), treatment was continued for an additional 2 months for a total treatment period of 4 months (Mo4). At baseline and each month thereafter, a venous blood sample was obtained after an overnight fast, along with a review of systems to identify any change from baseline status. The blood specimen was sent to a commercial laboratory (LabCorp, Raritan, NJ, USA) for evaluation of the serum lipoprotein profile [e.g., HDL-C, LDL-C, very low-density lipoprotein cholesterol (VLDL-C), TG, total cholesterol (TC)], glucose, insulin, LFTs, eGFR), and a complete blood count with differential (CBC w/diff). All blood sample results were reviewed by the study physician for the presence of adverse findings. The presence of an adverse finding in LFTs, kidney function, CBC w/diff, or a change in the patient self-report health status were submitted to the Institutional Review Board (IRB) as an adverse event; the nature/severity of the event may have resulted in discontinuation of the subject from the study drug and continued participation in the trial, which was contingent upon the judgement of the study physician and guidance from the IRB. All participants were instructed to continue their normal diet and patterns of physical activity while participating in the study. At the end of each month, participants in the treatment group were required to return any unused study drug that may have resulted from a missed dose. No medications were returned by any subject, and the investigators were, therefore, working under the assumption that all participants were 100% compliant with the prescribed drug treatment.

### Statistical analyses

Values are expressed as group mean ± SD, unless otherwise indicated. Separate analysis of variance (ANOVA) were performed to identify group differences (i.e., control, treatment) for baseline demographic data [e.g., age, height, weight, BMI, duration of injury (DOI)]. Pearson chi-square tests were performed to identify differences for the number of enrolled participants in each group based on gender (e.g., male, female), injury level (e.g., paraplegia, tetraplegia), AIS level (e.g., A, B, C), and ethnicity (e.g., African American, White, Hispanic). Two control subjects missed a follow-up visit and the outcome measurements from the previous visit were carried forward to facilitate the statistical analyses. Separate 2 (group: treatment, control) X 3 (visit: baseline, Mo2, Mo4) mixed-model repeated measures ANOVA (RMANOVA) were performed to determine if differences were present for serum lipoprotein profile [e.g., HDL-C, LDL-C, VLDL-C, TG, TC], glucose, insulin, and the TG/HDL-C, LDL-C/HDL-C and TC/HDL-C ratios. Separate factorial ANOVA were performed to identify the presence of group differences in the percent change from baseline to 2 and 4 months, respectively in the serum TG, HDL-C, and LDL-C. The nature of significant group or time main effects were explored with Tukey *post hoc* tests. Although not part of the primary analysis, results from the venous blood samples at months 1 and 3 visits are provided. Statistical analyses were completed using IBM SPSS Statistics 25 (IBM, Armonk, NY) and figures were created with GraphPad Prism (version 8.0 for Windows, GraphPad Software, San Diego, CA). An *a priori* level of significance was set at p ≤ 0.05.

### Statement of ethics

The research protocol was approved by the IRBs of the host institutions. Written informed consent was obtained from each subject prior to study participation. All applicable institutional and governmental regulations concerning the ethical use of human volunteers were followed during this research study. The trial was first registered in May 27, 2015 on ClinicalTrials.gov under the number: NCT02455336.

### Ethics apprival and consent to participate

The research protocol was approved by the Institutional Review Board of the James J. Peters VA Medical Center (Bronx, NY) and the Kessler Institute for Rehabilitation (West Orange, NJ). Written informed consent was obtained from each subject prior to study participation. All applicable institutional and governmental regulations concerning the ethical use of human volunteers were followed during this research study. The trial was registered on ClinicalTrials.gov under the number: NCT02455336.

## Data Availability

The datasets generated and/or analyzed during the current study are not publicly available because analyses of the outcomes remain on-going, but are available from the corresponding author on reasonable request.
